# Physicochemical Characteristics of Transferon*™* Batches

**DOI:** 10.1155/2016/7935181

**Published:** 2016-07-20

**Authors:** Emilio Medina-Rivero, Luis Vallejo-Castillo, Said Vázquez-Leyva, Gilberto Pérez-Sánchez, Liliana Favari, Marco Velasco-Velázquez, Sergio Estrada-Parra, Lenin Pavón, Sonia Mayra Pérez-Tapia

**Affiliations:** ^1^Unidad de Desarrollo e Investigación en Bioprocesos (UDIBI), Escuela Nacional de Ciencias Biológicas, Instituto Politécnico Nacional, Prolongación de Carpio y Plan de Ayala s/n, Colonia Santo Tomás, 11340 Ciudad de México, Mexico; ^2^Departamento de Farmacología, Cinvestav-IPN, Avenida Instituto Politécnico Nacional 2508, Colonia San Pedro Zacatenco, 07360 Ciudad de México, Mexico; ^3^Laboratorio de Psicoinmunología, Dirección de Investigaciones en Neurociencias, Instituto Nacional de Psiquiatría Ramón de la Fuente, Calzada México-Xochimilco 101, Colonia San Lorenzo Huipulco, 14370 Ciudad de México, Mexico; ^4^Departamento de Farmacología, Facultad de Medicina, Universidad Nacional Autónoma de México, Ciudad Universitaria, 04510 Ciudad de México, Mexico; ^5^Departamento de Inmunología, Escuela Nacional de Ciencias Biológicas, Instituto Politécnico Nacional, Prolongación de Carpio y Plan de Ayala s/n, Colonia Santo Tomás, 11340 Ciudad de México, Mexico; ^6^Unidad de Investigación, Desarrollo e Innovación Médica y Biotecnológica (UDIMEB), Escuela Nacional de Ciencias Biológicas, Instituto Politécnico Nacional, Prolongación de Carpio y Plan de Ayala s/n, Colonia Santo Tomás, 11340 Ciudad de México, Mexico

## Abstract

Transferon, a biotherapeutic agent that has been used for the past 2 decades for diseases with an inflammatory component, has been approved by regulatory authorities in Mexico (COFEPRIS) for the treatment of patients with herpes infection. The active pharmaceutical ingredient (API) of Transferon is based on polydispersion of peptides that have been extracted from lysed human leukocytes by a dialysis process and a subsequent ultrafiltration step to select molecules below 10 kDa. To physicochemically characterize the drug product, we developed chromatographic methods and an SDS-PAGE approach to analyze the composition and the overall variability of Transferon. Reversed-phase chromatographic profiles of peptide populations demonstrated batch-to-batch consistency from 10 representative batches that harbored 4 primary peaks with a relative standard deviation (RSD) of less than 7%. Aminogram profiles exhibited 17 proteinogenic amino acids and showed that glycine was the most abundant amino acid, with a relative content of approximately 18%. Further, based on their electrophoretic migration, the peptide populations exhibited a molecular mass of about 10 kDa. Finally, we determined the Transferon fingerprint using a mass spectrometry tool. Because each batch was produced from independent pooled buffy coat samples from healthy donors, supplied by a local blood bank, our results support the consistency of the production of Transferon and reveal its peptide identity with regard to its physicochemical attributes.

## 1. Introduction

Transferon, a human dialyzable leukocyte extract (DLE), is obtained by disrupting the cells from buffy coats, followed by a dialysis step using a 12 kDa cutoff membrane. The permeated product of 12 kDa is then subjected to ultrafiltration to obtain molecules below 10 kDa [1, 2]. Transferon is a biotherapeutic agent that has been approved by the Mexican health authorities for human consumption as an immunomodulator [1, 2]; thus, continuous characterization and analysis of this agent are increasing our knowledge about its properties, supporting the quality of this product.

The complex mixture of small peptides from DLEs, also known as transfer factor, has therapeutic effects on a wide range of diseases with an inflammatory component [3], such as infections [4], autoimmune diseases [5], immunodeficiencies [6], allergies [7], and cancer [8, 9]. Although DLEs have more than 50 years of clinical history as a treatment for several diseases [10], regulations concerning biological products and the use of pharmaceutical peptides have evolved significantly in recent years. Further, a concerted effort has been made to determine their peptide composition. Several methods have been reported in attempt to determine the structure and composition of DLEs from various species [2, 11–13], such as a description of the amino acid content in pig and rabbit spleen-derived dialyzable extracts [14, 15], as well as bursa-derived dialyzable extracts [12]; a conserved sequence has even been determined in DLEs from mice and calves [11, 16].

These types of biotherapeutic products are regarded as alternative options in the treatment of immune and infectious diseases, based on their high bioactivity, low toxicity, and negligible tissue accumulation [17, 18]. Conversely, such agents are complex and exhibit batch-to-batch variability, limiting the reproducibility of these studies, and their full physicochemical characterization is a challenge for analytical chemistry and quality control. Consequently, the methods for describing the active pharmaceutical ingredients (APIs) in peptide-derived pharmaceutical products, such as Transferon, must include several analytical steps that can distinguish all of the components in a sample in its native form or after treatment [14, 19, 20].

APIs are well regulated as per the criteria of worldwide health regulatory agencies [21]. The physicochemical characterization of biological products is a crucial step in determining their composition to establish the critical quality attributes that might impact their safety and efficacy during their life cycle [22].

Electrophoresis and liquid chromatography are 2 of the most suitable techniques for analyzing peptides and proteins, the former of which is one of the most versatile analytical techniques available. Reversed-phase ultrahigh-performance liquid chromatography with ultraviolet detection (RP-UHPLC-UV) enables the characterization of complex samples based on the hydrophobicity of their components [23], generating a peptide mapping profile according to their polarity. UHPLC also allows one to determine the amino acid composition of a sample after acidic digestion [24] and, when coupled to a mass detector, the intact mass of its components [25].

The API in Transferon has been described as a polydisperse mixture of peptides by size exclusion ultrahigh-performance liquid chromatography (SE-UHPLC) [2]; nevertheless, the peptide identity and amino acid composition of this product have not been published. The aim of this study was to physicochemically characterize the peptide and amino acid content of 10 batches of Transferon using SDS-PAGE and chromatographic techniques to establish the quality and overall variability of its API.

## 2. Materials and Methods

### 2.1. Analytical Samples

Transferon batches were manufactured using a modified version of Borkowsky and Lawrence's method [26], employing human leukocyte concentrates that were obtained from a certified blood bank as raw material. Briefly, more than 500 human leukocyte concentrates were subjected to several freeze-thaw cycles, wherein, in each cycle, the concentrate was frozen at −20°C for 1 week using a biomedical freezer (Sanyo, Osaka, Japan) and then thawed completely. Immediately after the last cycle, the leukocyte concentrates were pooled and dialyzed using a membrane with 12 kDa cutoff (Sigma-Aldrich, Missouri, USA). The dialyzed product was ultrafiltered using a SartoJet ultrafiltration system (Sartorius, Goettingen, Germany) with a 10 kDa cutoff cartridge to obtain molecules below 10 kDa.

Transferon samples were obtained from 10 typical batches (14E14, 14F16, 14F17, 14G18, 14G19, 14M27-A, 14M27-B, 14M28, 15A01, and 15A02), produced by UDIMEB at good manufacturing practices facilities as previously described. All Transferon batches in this study fulfilled the safety and efficacy criteria of Mexican health authorities (COFEPRIS) and UDIMEB. These quality control steps included (a) measurement of endotoxin levels by Endosafe Portable Test (Charles River Laboratories, Charleston, SC, USA) as per the manufacturer's instructions and Mexican Pharmacopoeia [27]; (b) a microbiological test as per Mexican Pharmacopoeia [28]; (c) determination of final peptide content by BCA method [29]; (d) identity test, comprising the comparison of the mass distribution profile by SE-UPLC between an internal standard of Transferon and typical batches [2]; and (e) measurements of biological activity using a murine model of cutaneous HSV-1 infection [1].

### 2.2. Peptide Quantification

Peptide concentration was measured by BCA method using the Pierce*™* BCA kit (Thermo Fisher Scientific, Massachusetts, USA) as per the manufacturer's instructions and a solution of bovine serum albumin as standard for the calibration curve [29]. Samples were analyzed on an EPOCH spectrophotometer (BioTek, Vermont, USA) using Gen5 software (BioTek).

### 2.3. Reversed-Phase Chromatography

Hydrophobic and hydrophilic characteristics of the peptide components of Transferon were analyzed by reversed-phase UHPLC (RP-UHPLC). All samples were filtered through a 0.10 *µ*m polyvinylidene fluoride (PVDF) membrane (Merck Millipore Co., Darmstadt, Germany) before being injected into the UPLC system. Chromatographic runs were performed on an H Class Acquity*™* UPLC system (Waters, Massachusetts, USA) that was equipped with a tunable UV/VIS detector (Waters).

Chromatographic separation was performed using an Acquity UPLC BEH300 C18 chromatographic column (1.7 *μ*m, 2.1 mm × 150 mm) (Waters) with gradient workflow of 0.4 mL/min of ultrapure purified water (Merck Millipore Co., Darmstadt, Germany) with 0.1% trifluoroacetic acid (TFA) (Sigma-Aldrich, Missouri, USA) and acetonitrile (Mallinckrodt Baker, Pennsylvania, USA) and 0.1% TFA (Sigma-Aldrich). The gradient began with 100% aqueous TFA and decreased gradually to 70% from 2 to 12 min. Then, aqueous TFA was returned to 100% from 12 min to the end of the analysis (20 min). The column temperature and sample cooler were maintained at 30°C and 8°C, respectively. The UV/VIS detector was set to 214 nm for data acquisition, and the analysis was performed using Empower (Waters). Only chromatographic peaks with a capacity factor (*k*) higher than 1.0 were considered for analysis.

### 2.4. Amino Acid Analysis

The amino acid composition of Transferon was determined by acid hydrolysis of the entire sample by RP-UPLC analysis. Twenty microliters of Transferon was hydrolyzed with 180 *µ*L 6 N hydrochloric acid (Thermo Scientific, Illinois, USA) for 18 h at 110°C under anaerobic conditions. The hydrolyzed samples were recovered in a 1.5 mL vial and dehydrated on Savant Speed Vac*™* (Thermo Scientific).

The samples were resuspended in 70 *µ*L borate buffer, pH 9.0, and 20 *µ*L 10 mM derivatizing reagent (6-aminoquinolyl-N-hydroxysuccinimidyl carbamate, AQC) as per instructions for the AccQ-Tag*™* Ultra application (Waters) [30], vortexed gently, and incubated for 10 min at 55°C. Individual amino acids were identified by UPLC analysis. Derivatized Transferon samples were injected into an Acquity UPLC system (Waters) using an Ultra C18 chromatographic column (1.7 *µ*m, 2.1 × 100 mm) (Waters) with gradient workflow of 0.7 mL/min of acetonitrile-formic acid-water, as per the manufacturer's instructions [30]. Both eluents were obtained from Waters. The temperatures for the column and autosampler were set to 43°C and 20°C, respectively. The detection was performed at 260 nm on a UV/VIS detector (Waters). We identified individual amino acids by comparing the amino acid chromatographic profiles (aminogram) of Transferon samples with an aminogram of a standard mixture of 50 pmol/*µ*L amino acid hydrolysate (Waters). We also analyzed a sample matrix blank and reagent blank as controls. The standard amino acid mixture and blanks were analyzed using the same method and under the same conditions as described for the Transferon samples.

### 2.5. SDS-PAGE

We determined the electrophoretic profile of the peptide components of Transferon under denaturing and nonreducing conditions. Transferon samples were concentrated by lyophilization using the FreeZone Freeze Dry System (Labconco, Missouri, USA). Lyophilized samples were reconstituted in ultrapure water (Millipore, Massachusetts, USA) and Laemmli sample buffer (Bio-Rad, California, USA) (1 : 1 ratio). Then, 15 *µ*L of each sample was loaded onto 16% polyacrylamide gels. We used equine myoglobin (17 kDa) (Bio-Rad) and a protein standard (2–250 kDa) (Bio-Rad) as molecular weight references.

Electrophoresis was performed at 80 V for 2.5 h using a Tris-Glycine buffer system (Bio-Rad), and the separated peptides were visualized by silver nitrate staining. Gel images were acquired on a ChemiDoc*™* system (Bio-Rad), and the data were analyzed using Image Lab*™* software (Bio-Rad).

### 2.6. Mass Spectrometric Analysis

Peptide composition and homogeneity of Transferon batches were determined by MS (mass spectrometry) using an ESI-Q-TOF (electrospray ionization-quadrupole-time of flying) mass detector, coupled to an Acquity chromatographic system (Waters). Transferon samples were filtered through a 0.10 *µ*m PVDF membrane (Merck Millipore Co.), and 5 *μ*L was injected into the chromatographic system. Sample desalination and separation were performed on a BEH C8 column (130 Å, 1.7 *μ*m, 2.1 × 100 mm) (Waters) using aqueous formic acid (0.1%) (Sigma-Aldrich) (Solution A) and acetonitrile (Mallinckrodt Baker) (Solution B). Column temperature was maintained at 30°C, and the flow rate was set at 0.2 mL/min using a gradient configuration (1-2 min, 60% A; 3–40 min, 90% A; 41–45 min, 60% A). Mass spectrometric analysis was performed on a Xevo G2-SQ-TOF mass detector (Waters). Electrospray mass spectra data were recorded in a positive ion mode for a mass range from 100* m/z* to 2000* m/z* with a scan time of 0.1 s. The source temperature was set at 150°C with a cone gas flow of 20 L/h. Desolvation gas flow was set at 600 L/h and 250°C. The capillary was maintained at 3.5 kV, and the cone voltage was set to 20 V. Mass spectra data were analyzed using Mass Lynx*™* (Waters).

### 2.7. Statistical Analysis

All experiments were performed using 10 Transferon batches (*n* = 10), except for the mass spectrometric analysis (*n* = 5). In determining the homogeneity of peptide components between Transferon batches, we calculated the mean, standard deviation, and relative standard deviation (% RSD) for the chromatographic peaks in the RP-UPLC analysis. In the amino acid composition assay, we also estimated the average relative abundance of proteinogenic amino acids and the standard error (SE) of the mean.

## 3. Results and Discussion

Transferon is a human DLE that is manufactured in good manufacturing practices facilities and has been approved for human use by Mexican health regulatory agencies. The biological activity of this biotherapeutic agent with immunomodulatory properties is associated with its peptide polydispersion profile in the treatment of diseases with an inflammatory component [1, 3, 31]. Although Transferon is generated from peripheral blood lymphocytes of healthy human donors, its molecular size distribution is consistent from batch to batch by SE-UPLC [2].

Certain biodrugs that are obtained from biological extraction are too complex to fully characterize, given their heterogeneous composition [32, 33]. For such drugs, sensitive and reliable analytical methods and robust manufacturing processes require quality specifications and in-process controls, respectively, to ensure their safety and efficacy between batches. Because Transferon is a mixture of peptides, it requires in-depth characterization to provide physicochemical evidence of its composition [2]. In this study, we used well-known analytical techniques, including chromatographic, electrophoretic, and mass spectrometric analyses, to characterize Transferon physicochemically.

### 3.1. Peptide Hydrophobicity

**Figure 1:**
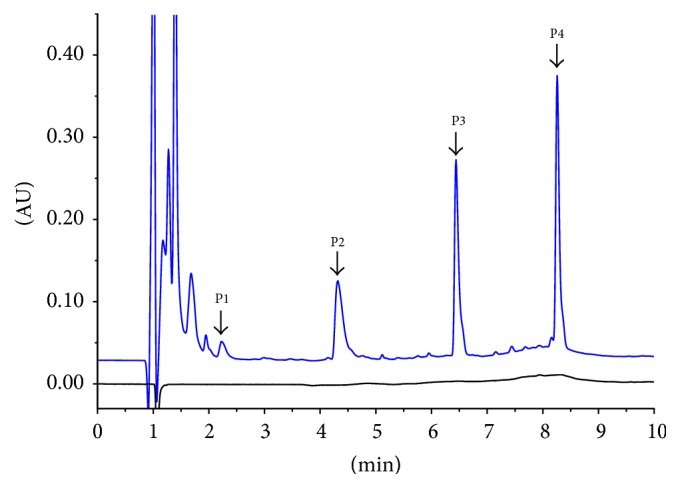
Reversed-phase chromatographic profile of Transferon. Comparison between sample matrix (black line) and batch 15A01 of Transferon (blue line). Chromatographic profile exhibits 4 main peaks with *k* > 1 and absolute retention time of 2.2 min (P1), 4.3 min (P2), 6.4 min (P3), and 8.2 min (P4). Figure S1 shows that this chromatographic profile is consistent between the 10 analyzed Transferon batches. Transferon samples were analyzed using an Acquity*™* UPLC*™* BEH300 C18 chromatographic column (2.1 mm × 150 mm) and TFA (0.1%)-H_2_O and TFA (0.1%)-acetonitrile as the mobile phase at 0.4 mL/min using a gradient configuration. The column temperature was maintained at 30°C, and UV detection was performed at 214 nm. Chromatographic profiles were analyzed using Empower*™* (ApexTrack method) to obtain the relative area percentage and absolute retention time for each peak. AU: area units.

DLEs are composed of very poorly hydrophobic peptide populations, which is challenging for analysis by RP-HPLC [11]. Using TFA as an ion pair, we designed a simple RP method that could identify 4 main chromatographic peaks (*k* > 1) in Transferon samples with absolute retention time of 2.2 min (P1), 4.3 min (P2), 6.4 min (P3), and 8.2 min (P4) (Figure 1 and Table S1 (in Supplementary Material available online at http://dx.doi.org/10.1155/2016/7935181)). After analyzing the 10 batches (Figure S1), we determined the average percentage area of each peak to be 3.87%, 25.33%, 31.57%, and 39.24%, respectively, with high reproducibility (RSD less than 7.0%) (Table 1).

As shown in Figure 1, chromatographic profile of Transferon also shows 6 peaks with retention time lower than 2 min and, as consequence, a *k* value lower than 1. This means that these molecules that elute close to the void volume are highly hydrophilic entities; therefore, the interaction with the stationary phase is absent or weak and the separation process is inefficient; these peaks were excluded from the analysis. However, this examination contributes to the comprehensive knowledge of a complex peptide mixture, such as Transferon.

These results show that although the precise identity of the peptide populations in Transferon remains unknown, their hydrophobic characteristics are consistent. Taking into account the hydrophobicity and the previously determined molecular weight distribution of Transferon components [2], our findings demonstrate that the proportion and physicochemical nature of Transferon components are highly reproducible between batches.

### 3.2. Amino Acid Composition

**Figure 2:**
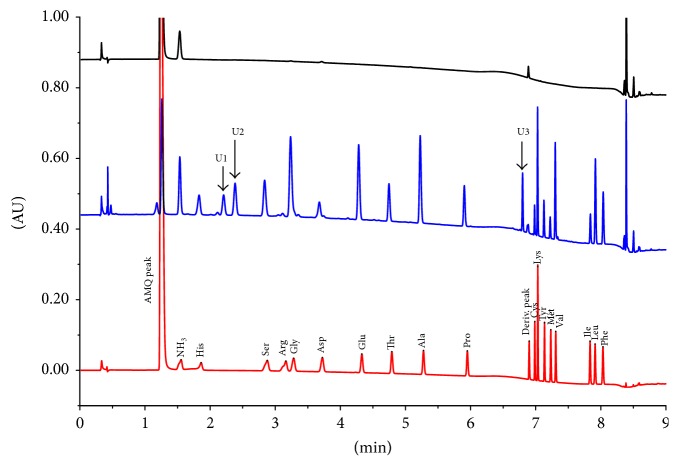
Amino acid chromatographic profile (aminogram) of Transferon. Comparison between standard mixture of hydrolyzed proteinogenic amino acids (Waters*™*) (red line) and batch 15A02 of Transferon (blue line). A sample matrix derivatization control is also shown (black line). A total of 17 of the 20 known proteinogenic amino acids were detected using the amino acid standard. The amino acid chromatographic profile also showed three unidentified peaks, which were labeled as U1 (2.25 min), U2 (2.37 min), and U3 (6.80 min). Figure S2 evinces the notion that the aminogram profile is consistent between the 10 Transferon batches. The samples were analyzed using an Acquity C18 column (1.7 *µ*m, 2.1 × 100 mm) with a mixture of acetonitrile-formic acid-water as the mobile phase using a gradient configuration. The column was maintained at 43°C, and UV detection was monitored at 260 nm. AMQ, NH_3_, and deriv. peaks originate from the reaction of derivatization. AU: area units.

**Figure 3:**
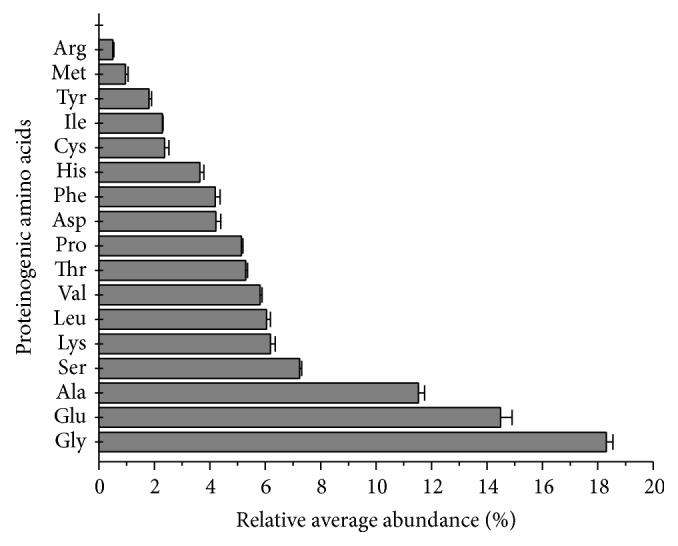
Relative average abundance of proteinogenic amino acids in 10 Transferon batches. Gly (18.30%), Glu (14.49%), and Ala (11.53%) are the most abundant amino acids in Transferon samples, whereas Met (0.95%) and Arg (0.50%) are the least abundant. Total area of the chromatographic peaks of the identified proteinogenic amino acids was considered 100% for calculations of abundance.

To determine the peptide composition of the chromatographic peaks in the molecular size exclusion and hydrophobic chromatographic profiles of Transferon, we analyzed its amino acid residues. The aminograms of Transferon corresponded to the amino acid standards (Figure 2). This comparison allowed us to identify 17 proteinogenic amino acids in the Transferon samples, the most abundant of which was Gly (18.30%); Arg was the least abundant (0.50%) (Figure 3). This analysis was not able to identify the abundance of Gln and Asn, given that these amino acids are hydrolyzed during the analysis to Glu and Asp, respectively. The amino acid composition profile between 10 batches (Figure S2) and the relative average abundance of the 17 proteinogenic amino acids were highly reproducible, with an RSD of less than 5% and 25% for Gly and Arg, respectively. On the other hand, our amino acid analysis revealed the presence of 3 unidentified peaks (U1, U2, and U3) that accounted for 13.41% of the total area of the peaks (proteinogenic and unidentified amino acids) in the amino acid chromatographic profile (Figure 3). Taking into account the total area of the peaks, U1, U2, and U3 had relative average abundance of 2.86% (SE 0.31%), 6.56% (SE 0.46%), and 3.99% (SE 0.19%), respectively, indicating that although these nonproteinogenic amino acids remain unidentified, they are also consistent between batches.

Notably, our results are similar to the 2013 amino acid analysis of a rabbit-derived DLE by Zhou and colleagues [14], although the products were manufactured from different biological sources. The identification of individual amino acids in hydrolyzed Transferon samples confirms the peptide identity of the drug. Further, the low RSD values of amino acids between batches confirm that the peptide components of Transferon are highly reproducible.

### 3.3. Electrophoretic Analysis

**Figure 4:**
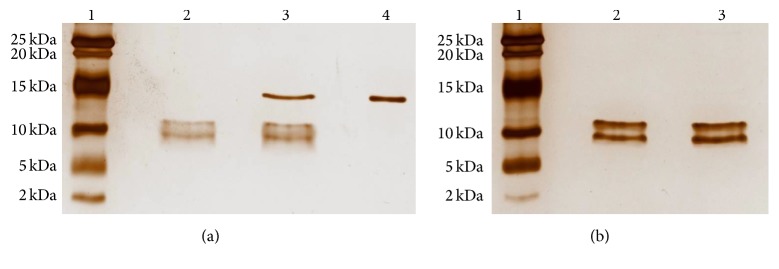
Electrophoretic profile of Transferon. (a)* Method selectivity*. The batch 14E14 of Transferon (85 *µ*g) showed 2 main bands at approximately 10 kDa (line 2). A second 14E14 sample (85 *µ*g) exhibited the same electrophoretic pattern when spiked with 5 *µ*g of equine myoglobin (17 kDa) (line 3). A total of 5 *µ*g of equine myoglobin was used as selectivity control (line 4). (b)* Batch reproducibility*. The electrophoretic patterns of batches 15A01 (line 2) and 15A02 (line 3) of Transferon (100 *µ*g) are consistent. Figure S3 shows the electrophoretic pattern of the 10 analyzed Transferon batches. Electrophoresis was performed in 16% acrylamide gels using a Tris-Gly system. Bands were detected by silver staining.

SDS-PAGE was performed to determine the molecular weight and electrophoretic migration of the peptide components in our product. The electrophoretic profile of Transferon samples comprised 2 bands at around 10 kDa and was selective for components of Transferon (Figure 4). Electrophoretic pattern was homogeneous between the 10 analyzed batches (Figure S3). Although the number of electrophoretic bands was less than expected, the electrophoretic profile of Transferon correlated with the molecular weights of the selected constituents during its manufacture (less than 10 kDa) and with a previously reported size exclusion chromatography analysis [2]. The inability of SDS-PAGE to detect the smaller peptide components of Transferon is due to its low resolution and sensitivity, compared with size exclusion chromatography. Notwithstanding, SDS-PAGE can be used as a straightforward primary identity test in DLE analysis.

### 3.4. MS-TOF Fingerprint

**Figure 5:**
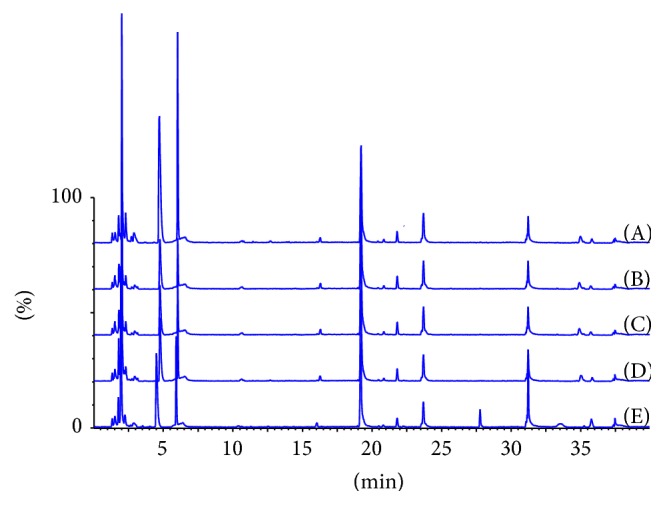
Peptide mapping of Transferon batches by MS-TOF. Five typical batches were subjected to spectrometric analysis and their TIC (total ion counting) profile did not exhibit significant differences. Transferon batches: 14E14 (A), 14F16 (B), 14F17 (C), 14G18 (D), and 14G19 (E).

Finally, we took advantage of the sensitivity of mass spectrometric analysis to obtain a fingerprint of Transferon. After RP-UHPLC desalination, 5 typical batches of Transferon were analyzed, and their total ion counting (TIC) profile spectrum showed 6 main peaks with retention time of 4.5 min, 5.5 min, 19.25 min, 21.75 min, 24.75 min, and 31.25 min (Figure 5). Batch (E) exhibited two additional peaks at 27.75 min and 33.50 min. This microheterogeneity was expected, considering the raw material from which Transferon is manufactured. Notwithstanding, these differences do not show impact on the biological effect of the product since each batch analyzed in this study accomplished the biological activity specification. This analysis is highly relevant, because the spectrometric fingerprint of a complex biological drug is useful for detecting differences between batches and between products and manufacturing processes.

The pharmacological activity of hDLEs has been associated with their peptide components, the primary sequence of which remains unknown. hDLEs exhibited high batch-to-batch variability, which compromises their study. We have developed an analytical platform that evaluates the batch-to-batch variability of hDLEs regarding the physicochemical properties of their peptide populations to diminish the variability between studies. This platform employs well-known analytical techniques (e.g., SDS-PAGE) and a sensitive MS-Q-TOF analysis, and this report is the first instance that a DLE has been subjected to an MS technique. Transferon shows high batch-to-batch homogeneity, even by MS analysis.

## 4. Conclusions

The analytical techniques that we used to physicochemically characterize Transferon have increased our knowledge about the peptide hydrophobicity, chemical composition, and molecular mass of the API. Collectively, these physicochemical analyses have revealed the identity of the product and confirmed the reproducibility and robustness of the manufacturing process in up to 10 typical batches. This research supports the quality of Transferon, an immunomodulator that has been widely used for more than 2 decades for the treatment of diseases with an inflammatory component, and provides a useful analytical platform to evaluate the variability between batches to improve the reliability of studies.

## Supplementary Material

Representative outcomes of each characterization assay are displayed in the main article, whereas the results of all analyzed Transferon samples are included in the supplementary material section in order to show the homogeneity between batches. The retention time (Table S1) and the chromatographic profile (Figure S1) in the reverse-phase analysis were highly consistent among 10 batches. Batch-to-batch reproducibility was also exhibited in the amino acid (Figure S2) and the electrophoretic assays (Figure S3).

## Figures and Tables

**Table 1:** Comparison of relative percentage areas of the 4 main peaks in the reversed-phase chromatographic profile of Transferon between 10 typical batches.

Batch	P1 (%)	P2 (%)	P3 (%)	P4 (%)
14E14	3.89	28.42	29.46	38.23
14F16	4.02	25.90	30.91	39.16
14F17	3.66	27.10	30.40	38.85
14G18	3.88	25.80	31.35	38.97
14G19	3.93	23.46	33.09	39.52
14M27-A	4.02	24.98	31.75	39.26
14M27-B	4.03	25.08	32.12	38.77
14M28	3.92	25.05	32.08	38.95
15A01	3.74	23.64	32.42	40.20
15A02	3.61	23.83	32.13	40.44

*Mean*	3.87	25.33	31.57	39.24
*% RSD*	3.91	6.19	3.38	1.70

## Abbreviations

API: Active pharmaceutical ingredient
BCA: Bicinchoninic acid
COFEPRIS: Comisión Federal para la Protección contra Riesgos Sanitarios
DLE: Dialyzable leukocyte extract
HSV-1: Herpes simplex virus type 1
SDS: Sodium dodecyl sulfate
PAGE: Polyacrylamide gel electrophoresis
RP-UHPLC-UV: Reversed-phase ultrahigh-performance liquid chromatography with ultraviolet detection
SE-UHPLC: Size exclusion ultrahigh-performance liquid chromatography
SE: Standard error.
