# Supplementary material for: Physicochemical Characteristics of Transferon™ Batches
Source: Biomed Res Int. 2016 Jul 20;2016:7935181. doi: 10.1155/2016/7935181 (PMC4971316; doi:10.1155/2016/7935181)
Supplement: Supplementary file 1 — Representative outcomes of each characterization assay are displayed in the main article, whereas the results of all analyzed Transferon samples are included in the supplementary material section in order to show the homogeneity between batches. The retention time (Table S1) and the chromatographic profile (Figure S1) in the reverse-phase analysis were highly consistent among 10 batches. Batch-to-batch reproducibility was also exhibited in the amino acid (Figure S2) and the electrophoretic assays (Figure S3). [file 7935181.f1.docx]

**Supplementary material**

**Physicochemical characteristics of Transferon™ batches**

Emilio Medina-Rivero, Luis Vallejo-Castillo, Said Vázquez-Leyva, Gilberto Pérez-Sánchez, Liliana Favari, Marco Velasco-Velázquez, Sergio Estrada-Parra, Lenin Pavón, and Sonia Mayra Pérez-Tapia.

***Table S1. Absolute retention time and k value of the chromatographic peaks detected in the RP-HPLC analysis of 10 Transferon™ batches.***

| **Batch** | | **Absolute Retention Time (min)** | | | |
| --- | --- | --- | --- | --- | --- |
|  |  | **Peak 1**  ***k = 1.2** | **Peak 2**  ***k = 3.3** | **Peak 3**  ***k = 5.4** | **Peak 4**  ***k = 7.2** |
| **1** | **14E14** | 2.256 | 4.373 | 6.453 | 8.259 |
| **2** | **14F16** | 2.261 | 4.365 | 6.448 | 8.262 |
| **3** | **14F17** | 2.251 | 4.362 | 6.449 | 8.265 |
| **4** | **14G18** | 2.254 | 4.363 | 6.448 | 8.262 |
| **5** | **14G19** | 2.248 | 4.345 | 6.439 | 8.262 |
| **6** | **14M27-A** | 2.259 | 4.381 | 6.448 | 8.259 |
| **7** | **14M27-B** | 2.261 | 4.385 | 6.441 | 8.259 |
| **8** | **14M28** | 2.245 | 4.343 | 6.447 | 8.262 |
| **9** | **15A01** | 2.235 | 4.317 | 6.442 | 8.260 |
| **10** | **15A02** | 2.231 | 4.317 | 6.434 | 8.261 |
| **Mean** | | **2.250** | **4.355** | **6.445** | **8.261** |
| **Std. Dev.** | | **0.010** | **0.024** | **0.006** | **0.002** |
| **%RSD** | | **0.461** | **0.552** | **0.086** | **0.023** |

* The value of k was obtained using the formula k = RT-RT_0_/RT_0_, where RT is the absolute retention time of each peak and RT_0_ is the “dead time” value or “void volume” (1 min).


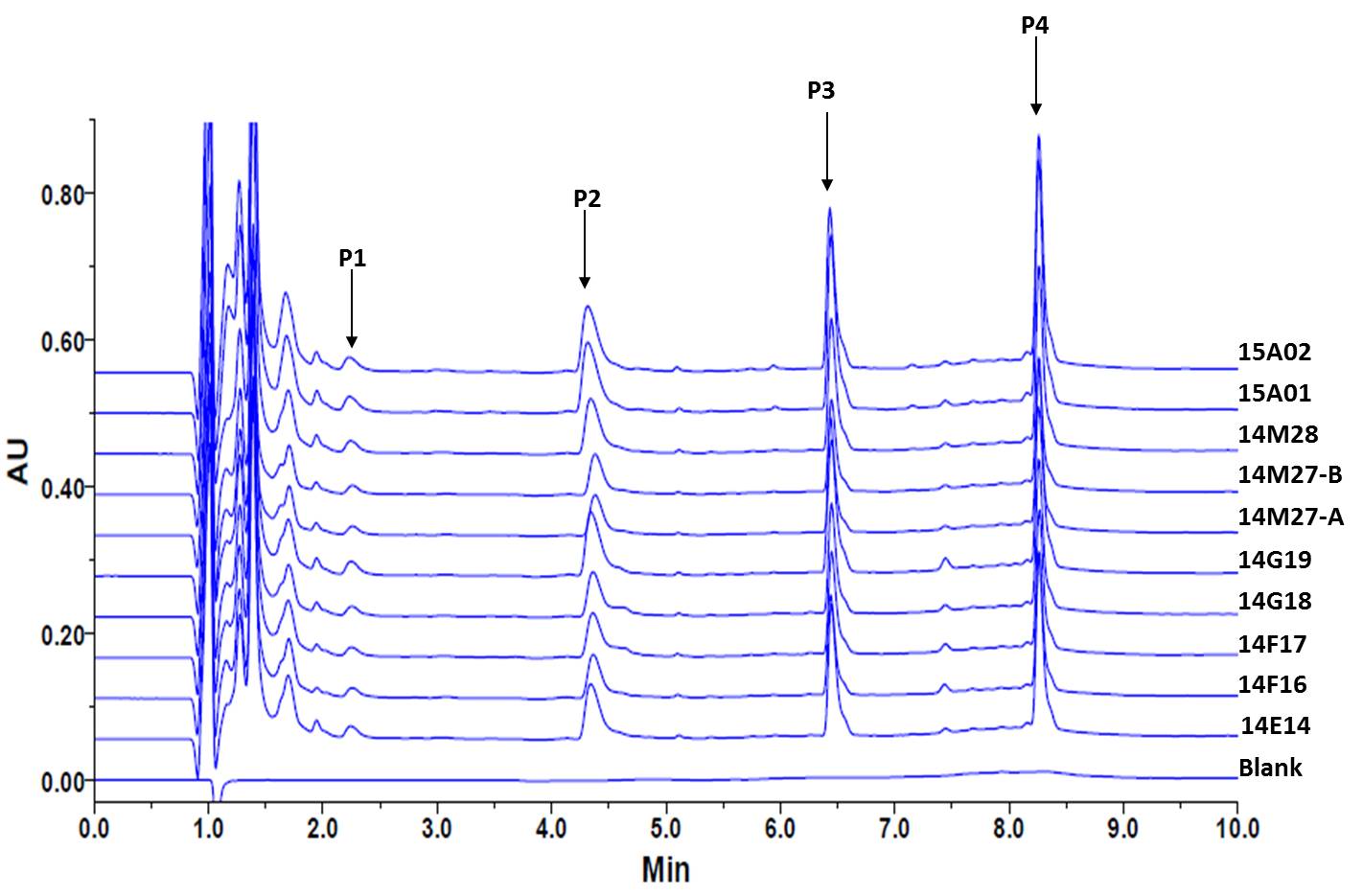


**Figure S1. Reverse-phase chromatographic profile of 10 Transferon™ batches.** Comparison between sample matrix and 10 Transferon™ batches. Chromatographic profile exhibits 4 main peaks (k > 1) with an absolute retention time of 2.2 min (P1), 4.3 min (P2), 6.4 min (P3) and 8.2 min (P4). Peaks with poor chromatographic separation (retention time lower than 2 min; k < 1) where excluded from the analysis. All samples were analyzed using an Acquity™ UPLC BEH300 C18 chromatographic column (2.1 mm x 150 mm) and TFA (0.1%)–H_2_O and TFA (0.1%)-Acetonitrile as the mobile phase at 0.4 mL/min using a gradient configuration. The column temperature was maintained at 30°C, and UV detection was performed at 214 nm. Chromatographic profiles were analyzed using Empower^TM^ (ApexTrack method) to obtain the relative area percentage and absolute retention time for each peak. AU; area units.


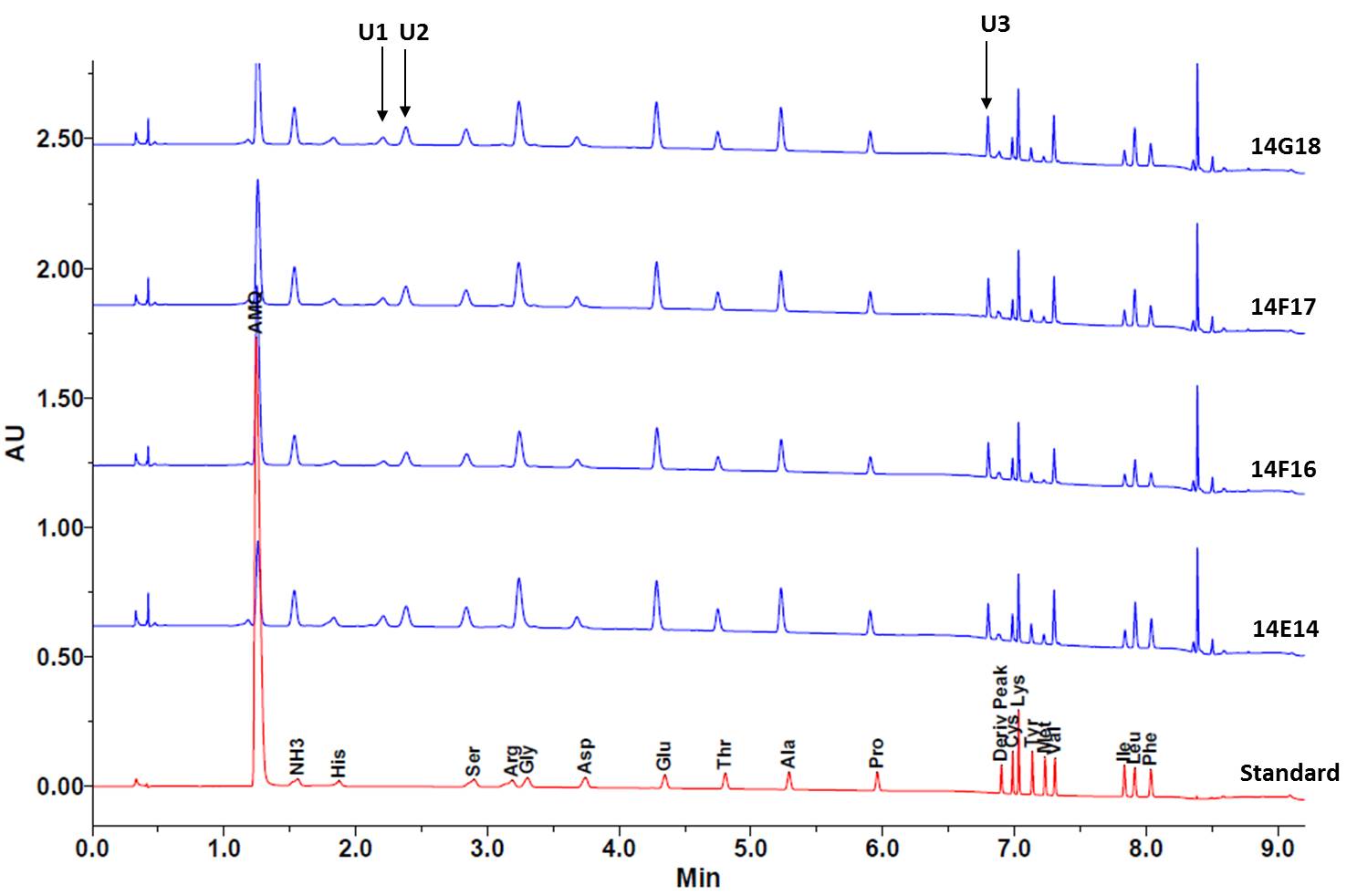
**A**


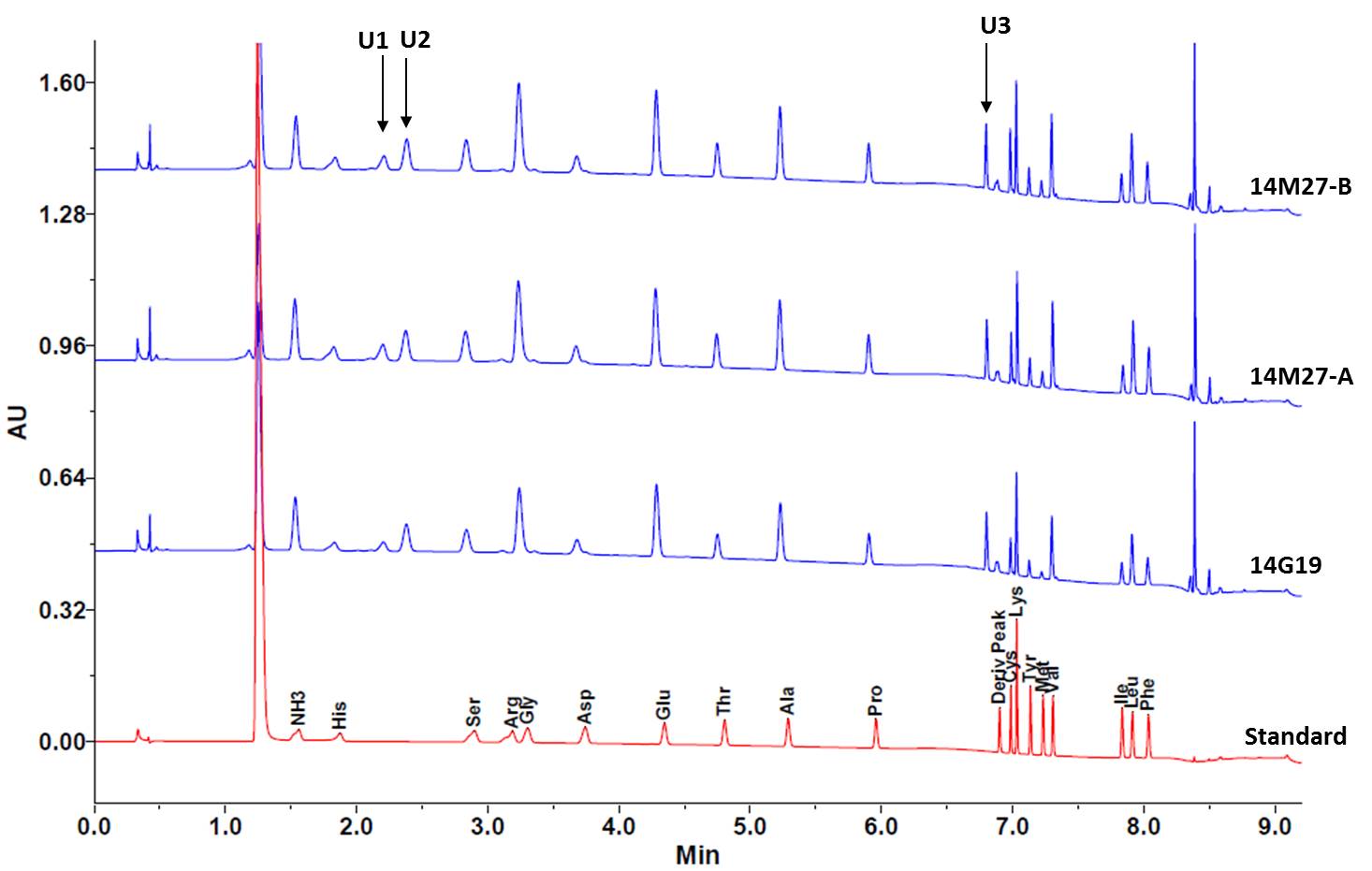


B


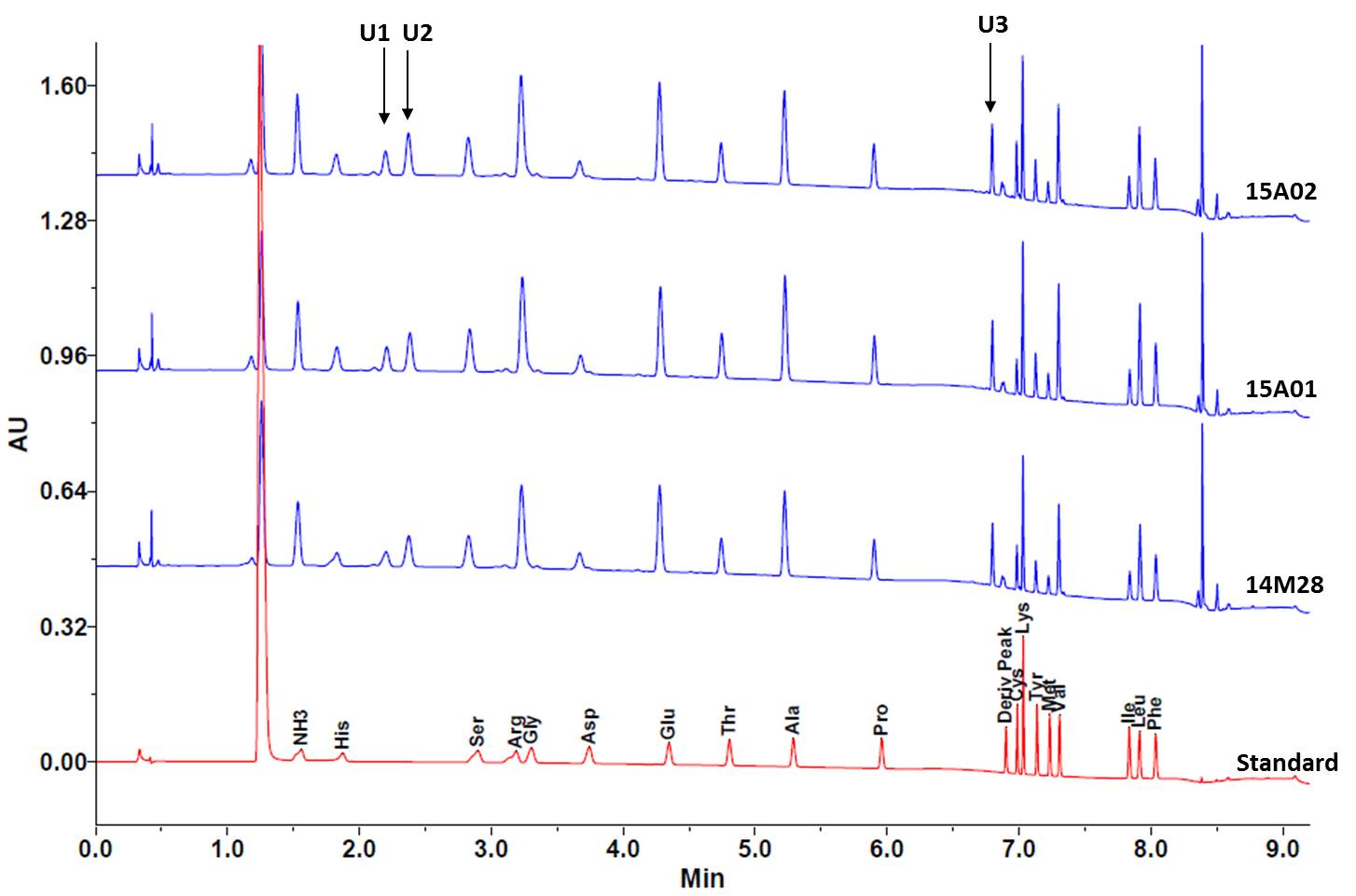
C

**Figure S2. Aminograms of hydrolyzed Transferon^TM^ samples from 10 different batches.** 10 amino acid profiles of different Transferon™ batches are shown from A to C; the profile of an amino acid standard mixture is also included in each image as reference. 17 out of the 21 observed peaks correspond to proteinogenic amino acids (His, Ser, Arg, Gly, Asp, Glu, Thr, Ala, Pro, Cys, Lys, Tyr, Met, Val, Ile, Leu and Phe). All Transferon™ samples exhibit 3 unidentified peaks: U1 (2.25 min), U2 (2.37 min) and U3 (6.80 min). The samples were analyzed using an Acquity™ C18 column (1.7 µm, 2.1x100 mm) with a mixture of acetonitrile-formic acid-water as the mobile phase using a gradient configuration. The column was maintained at 43°C, and UV detection was monitored at 260 nm. AMQ, NH_3_, and Deriv. peaks originate from the reaction of derivatization. AU; area units.


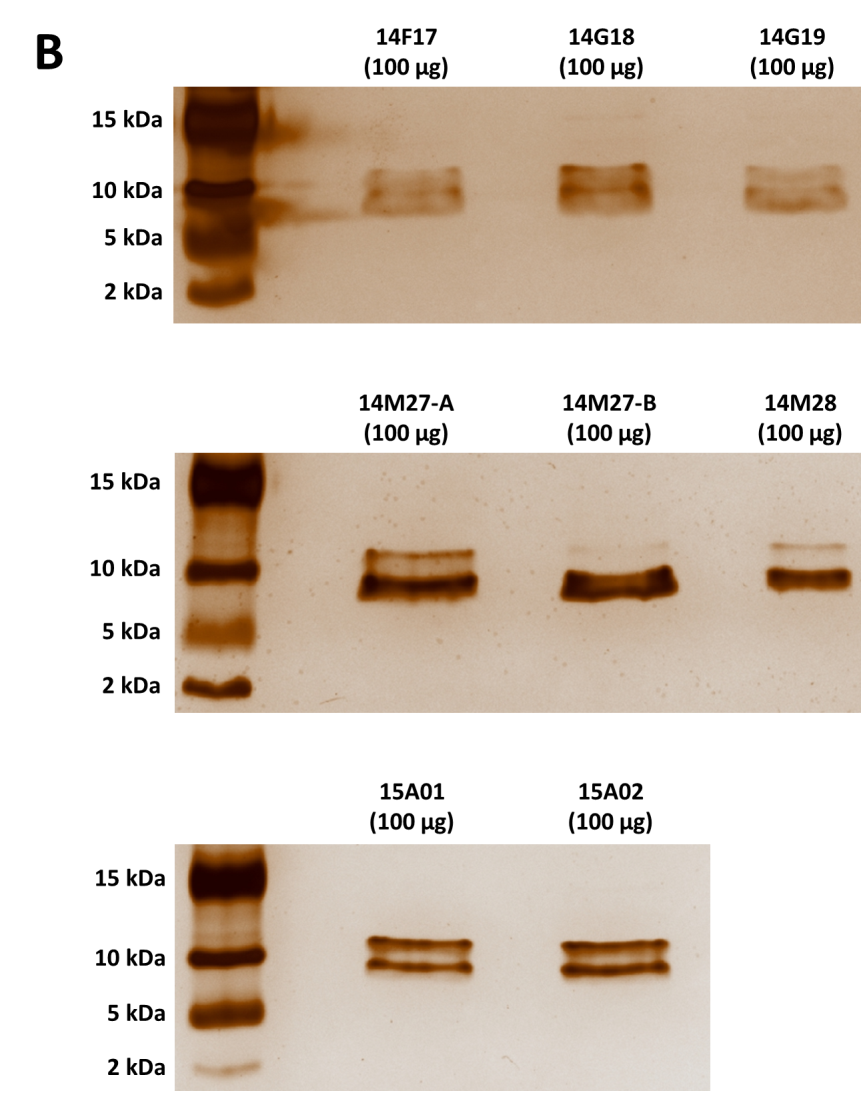


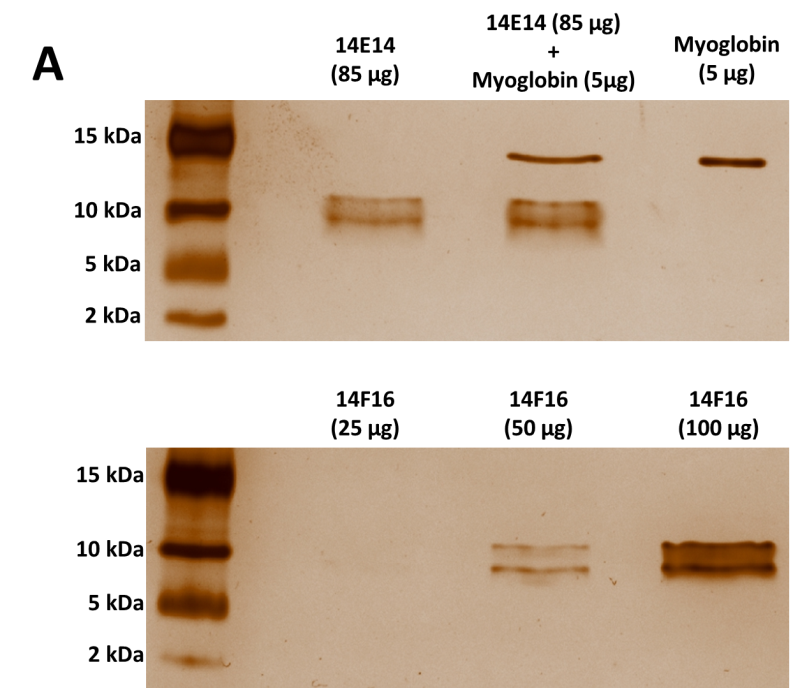

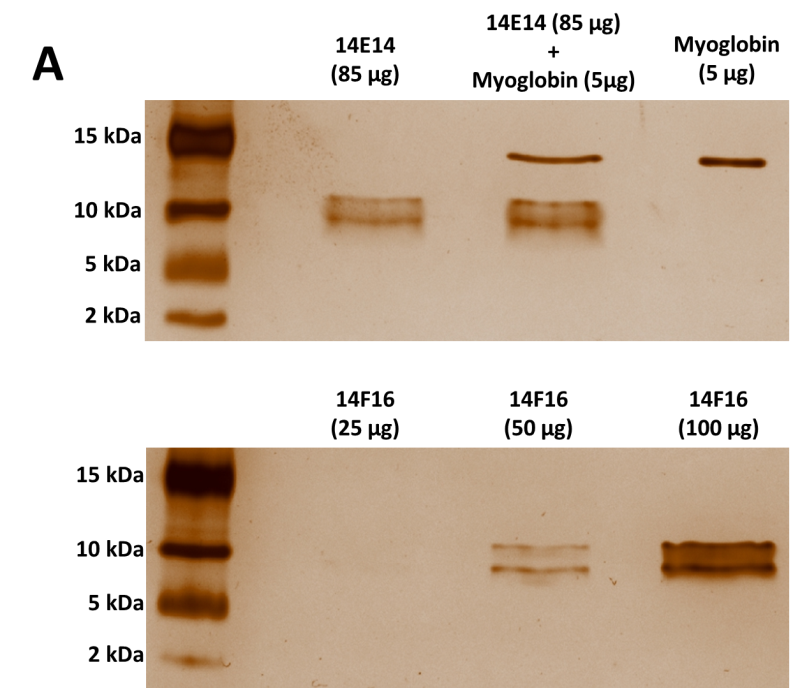


**Figure S3. Electrophoretic assay of 10 Transferon™ batches.** Two Transferon™ batches were employed to determine the selectivity (A, upper image) and the limit of detection (A, lower image) of the SDS-PAGE analysis; this assay is selective to Transferon™ components and exhibits a limit of detection of 50 μg. The electrophoretic profile of the two Transferon™ batches is characterized by two bands around 10 kDa (A), which was consistent between the rests of the 10 analyzed batches (B). Electrophoresis was performed in 16% acrylamide gels using a Tris-Gly system. Bands were detected by silver staining.
